# Skeletal muscle quantitative nuclear magnetic resonance imaging follow-up of adult Pompe patients

**DOI:** 10.1007/s10545-015-9825-9

**Published:** 2015-03-07

**Authors:** Pierre G. Carlier, Noura Azzabou, Paulo Loureiro de Sousa, Arnaud Hicks, Jean-Marc Boisserie, Alexis Amadon, Robert-Yves Carlier, Claire Wary, David Orlikowski, Pascal Laforêt

**Affiliations:** 1 Institut de Myologie and CEA, DSV, IBM, MIRCen, Laboratoire de RMN, Pitie-Salpetriere University Hospital, Bd de l’Hôpital, 75651 Paris Cedex 13, France; 2 ICube – IPB, Strasbourg University, Strasbourg, France; 3 CEA, DSV, IBM, Neurospin, UNIRS, Saclay, France; 4 Raymond-Poincaré University Hospital, Garches, France; 5 Centre de référence pour les maladies neuromusculaires de l’Est de Paris, Pitie-Salpetriere University Hospital, Paris, France

## Abstract

Adult late-onset Pompe disease is most often a slowly progressive limb-girdle and spine extensor muscle dystrophy, due to defective lysosomal acid maltase. With the exception of the few patients who present with a dramatically accelerated clinical course, standard diagnostic imaging fail to detect and evaluate disease progression between two successive visits. In muscle dystrophy of very rapid evolution, like the Duchenne disease, quantitative NMR imaging has successfully demonstrated its capacity to objectivate both disease activity and degenerative changes progression over short follow-up periods. The purpose of this retrospective monocentric open-label study was to investigate whether quantitative NMR imaging can monitor disease progression in adult Pompe patients despite its very slow nature. Quantitative imaging of Pompe patients succeeded in demonstrating that muscle fatty infiltration increased on average by 0.9 %/year, with the hamstring and adductor muscles showing the fastest degradation. Muscle water T2 mapping revealed that 32 % of all muscles had abnormally high T2 in at least one of two successive examinations. When muscle water T2 was abnormal, fatty degenerative changes were further increased by 0.61 %/year. Enzyme replacement therapy resulted in 0.68 %/year slowdown of the muscle fatty infiltration, in both muscles with normal and high T2s.

**Electronic supplementary material:**

The online version of this article (doi:10.1007/s10545-015-9825-9) contains supplementary material, which is available to authorized users.

## Introduction

Nuclear magnetic resonance (NMR) imaging has an established role in the diagnosis of inherited and metabolic muscle diseases (Wattjes et al 2010). In recent years, medical imaging has acquired a new dimension; it has become quantitative (Tofts 2003), the generation of parametric maps, in which voxel signal intensity is not in arbitrary units anymore but is the true reflection of a biological (% fat) or of an NMR (water T2) variable. For muscle disorders as for other ailments, the benefits of quantitative imaging are multiple: a better appreciation of disease severity, the possibility to monitor pathological changes over time and, most importantly, to evaluate skeletal muscle responses to therapeutic intervention. Quantitative variables and indices derived from NMR imaging but also spectroscopy constitute the most serious imaging candidates as biomarkers or outcome measures in clinical trials focusing on muscle pathology (Hollingsworth et al 2012).

The percentage of fat in muscle signal, commonly referred to as the percentage of fat, and the muscle water T2, not to be confused with the muscle global T2 (Carlier 2014), are currently the two NMR quantitative indices proposed to characterize a diseased muscle with imaging (Hollingsworth et al 2012). They convey very different information.

There exists several technical possibilities to separate water and fat signals in NMR images and, from these, to construct parametric maps of the percentage of fat signal in the images (Hu et al 2012). The percentage of fat in a chronically diseased muscle informs on the extent of the structural changes, by measuring the degree and extent of fatty involution of the damaged muscles. Water-fat imaging, using the Dixon technique, was able to measure the progression of the degenerative changes in thigh and lower leg muscles of limb-girdle muscular dystrophy (LGMD) 2I patients (Willis et al 2013). Adult form of Pompe disease is, in most patients, a disease of slow to very slow progression. Whether quantitative NMR imaging would be sensitive enough to pick up the small changes in muscle composition had to be ascertained.

Skeletal muscle cell T2, as more generally tissue water T2, is devoid of specificity and may be abnormal in a variety of pathological conditions. It is nevertheless an excellent indicator of the intensity of the underlying mechanisms and may be taken as a non-specific but sensitive indicator of “disease activity” (Tardif-de Géry et al 2000; Walter et al 2005; Thibaud et al 2007; Arpan et al 2013). Again it was valuable to determine whether quantitative T2 mapping would be able to evaluate disease activity in less aggressive forms of muscle dystrophy, like adult Pompe patients.

The benefit obtained by enzyme replacement therapy has been difficult to demonstrate unambiguously at least in adult Pompe patients (Güngör et al 2013). A further challenge for quantitative NMR imaging is to provide direct evidence of the effect of treatment on muscle structure and to gain acceptance as an outcome measure (Lachmann and Schoser 2013).

Muscle imaging, including quantitative sequences, has been part of the routine yearly follow up of the Pompe patients at the Institute of Myology, Paris for several years. Using this database, we decided to address several questions:

- Can quantitative water-fat imaging of skeletal muscle assess the progression of the fatty degenerative changes in muscles of adult Pompe patients?
- What degree of disease activity and what percentages of muscle involved does T2 mapping measure in the same patients?
- Is there a link between disease activity, as measured by T2 maps, and progression of the fatty degenerative changes in this population?
- Does enzyme replacement therapy have an effect on the progression of muscle damages in adult Pompe patients?

## Subjects/materials and methods

### Patients

Twenty-three patients, six males and 17 females, aged 22 to 69 years (49.0 ± 17.7 years), with a molecular diagnosis of Pompe disease, were retrospectively studied. All were followed at the Paris East Reference Center for Neuromuscular Disorders headed by Professor B. Eymard, Institute of Myology, Pitie-Salpetriere University Hospital, Paris. Population characteristics are given in a supplemental table.

Of the 23 patients, 14 were treated with enzyme replacement therapy (alglucosidase alfa, Myozyme®, Genzyme-Sanofi), for an average of 44 ± 20 months. The decision by the physicians in charge to treat was essentially taken according to disease severity. As a consequence, disease burden was more important on muscles of treated patients than on those of untreated ones and this prevented direct comparison of quantitative measurements between the two sub-groups.

Muscle imaging was part of the standard care provided to the Pompe patients at the Institute of Myology. All clinical data and complementary exams results were encoded in the French Pompe register. The natural history protocol of the untreated Pompe patients had received approval of the local ethics committee, as did alglucosidase alfa continuation agreement that most treated patients fell under.

For quantitative imaging, patients were examined in supine position, feet first.

### NMR equipment

Imaging was performed at 3 T on a Siemens Trio TIM machine, with elements of the spine receiver coils activated and three flexible “body” array coils covering anteriorly from ankles to hips.

### NMR imaging protocol

All patients were scanned at least twice at 1-year interval, 13 patients were examined three times and two were scanned during four consecutive years.

Standard T1w spin echo (SE) of the thigh and lower leg was first performed and muscle fatty degenerative changes were scored according to the Lamminen-Mercuri scale (grade 1: normal; grade 2: <30 %; grade 3: >30 % and <60 %; grade 4: >60 %).

Water-fat imaging of the entire lower limb was obtained with a modified Dixon sequence because of the unavailability of 3-point Dixon on Siemens machines. Two three-dimensional gradient echo (GE) volumes were acquired per segment, one with echo times (TE) of 2.75 and 3.95 ms, one with TEs of 2.75 and 5.15 ms, in order to collect the in-out-in images of a standard 3-point Dixon approaches. Slab volume was 448×448×320 mm, with a spatial resolution of 1×1×5 mm. The repetition time (TR) was 10 ms, with a flip angle of 3°, to ensure a proton-density weighting, and to be as close as possible to the hydrogen distribution in water and lipids. Amplitude and phase images were stored for water and fat image processing.

Quantitative T2 measurements were based on standard multi-slice multi echo time (TE) spin echo (SE) sequences. Seventeen echoes with an inter-echo spacing of 9.5 ms were acquired with a 3000 ms repetition time and an acceleration factor of 2. Two slabs of 11 slices, 10 mm thick, with 15 mm slice gap, were centered at mid-thigh and on the proximal third of the lower leg. In-plane resolution was 1.4×1.4 mm. No fat saturation module was activated.

Three-dimensional B1 mapping was collected with an optimized version of the actual flip angle imaging (AFI) sequence (Yarnykh 2007) provided by A. Amadon from Neurospin, CEA, France. The slabs encompassed the volumes scanned with the multiTE SE sequences.

Average scanning time was 90 min, in two sessions to include whole-body T1 weighted imaging which was performed head-first.

Altogether, 59 imaging sessions were reviewed, all but eight comprised both thighs and lower legs.

### Image analysis

**Fig. 1:**
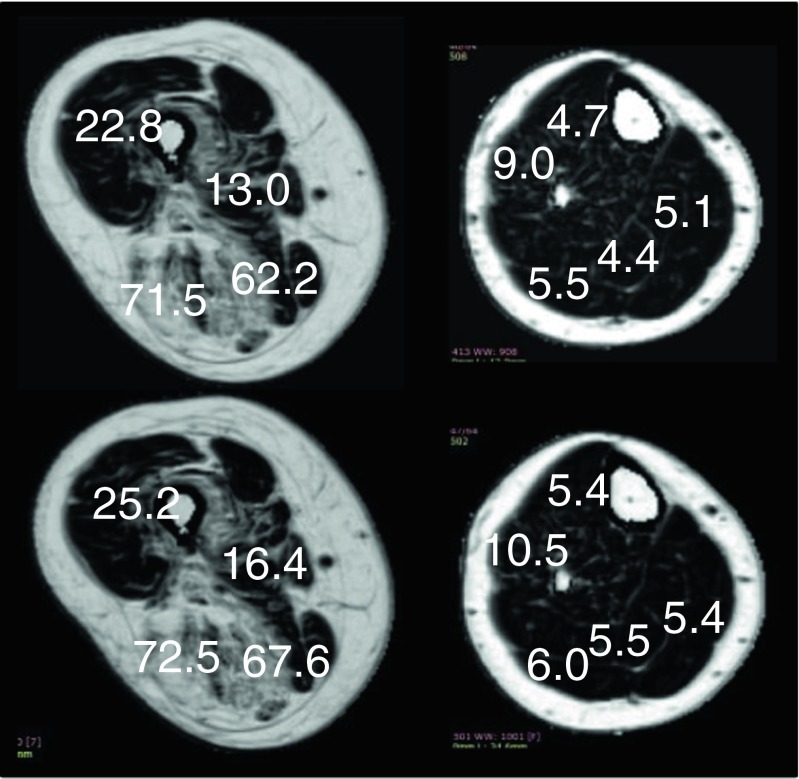
Examples of percentage of fat images generated from Dixon acquisitions in the thigh (*left*) and in the lower leg (*right*). The upper row was obtained at initial examination, the lower row show images at 1-year follow-up. The figures in white give the fat percentages in selected muscle — from top and clockwise, quadriceps, adducor longus, semi-membranous and biceps femoris, in the thigh; tibialis anterior, gasctrocnemius medialis, soleus, gastrocnemius lateralis and extensor digitorum, in the lower leg. In this example, the mean increase in fat infiltration was 2.9 % in the thigh and 0.9 % in the lower leg

Water, fat and percentage of fat volumes were reconstructed as previously described (Glover and Schneider 1991; Ma 2008) (Fig. 1).

Percentage of fat images were extracted at the same spatial coordinates as the multi-TE SE images and individual muscle fatty infiltration was measured.

Muscle water T2 values were calculated using a tri-exponential fitting procedure which allowed the separation from the fatty infiltrative component (Azzabou et al 2014). Threshold for abnormality was set at 2 standard deviation above mean muscle water T2 of volunteers, 39 ms.

### Statistics

Results are expressed as mean ± standard deviation.

Main variables, fat fraction, annual progression of fat fraction, and water T2 were analyzed using an analysis of variance (ANOVA) with between (treatment, high T2 vs normal T2) and within (muscle, segment, time) factors.

Linear relations between main variables were also determined.

## Results

### Yearly progression of muscle fatty degenerative changes in the lower limbs of Pompe patients

**Fig. 2:**
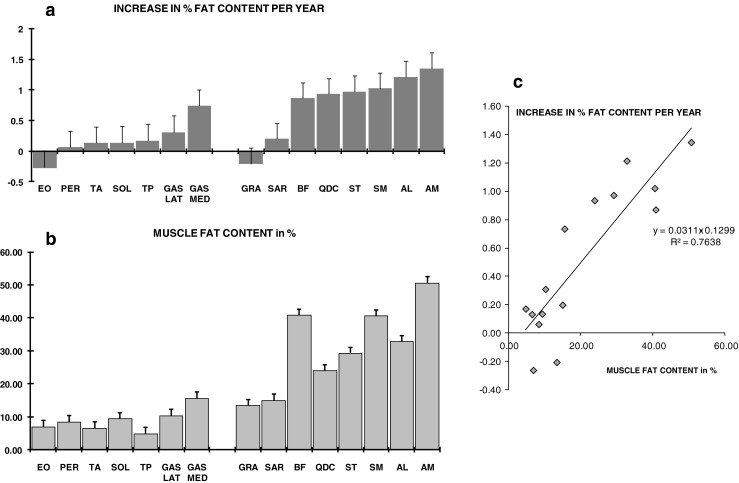
In A, the mean yearly increase in fat percentage is given muscle by muscle in the lower leg (*on the left*) and in the thigh (*on the right*). Using the same display, the degree of fatty degeneration is shown in B. There was a strong linear relationship between the extent of the fatty infiltrations and their progression as estimated by the annual increase in muscle fat content (see C)

Lamminen-Mercuri visual grading of T1w images of the thigh was 1.7 ± 1.0. In the lower leg, scores were close to normal (1.1 ± 0.3). No change in grading was noted over a 1-year observation period (0.0 ± 0.2). By contrast, the apparent fat content as defined in the Methods increased on average by 0.9 ± 0.2 %/year (*p* < 0.0001), all muscles of the lower limb considered. Individual muscle analysis, as displayed in Fig. 2, showed that intramuscular fatty degeneration progressed more rapidly in the thigh than in the lower leg, with the adductors longus and magnus experiencing the faster rate of fatty infiltration. In the lower leg, the gastrocnemius medialis was the only muscle for which fat content increased noticeably between exams. There was overall a very significant and logical correlation between the yearly rate of fatty replacement and the extent of the fatty degenerative changes. The muscles that were the most altered would be expected to be the ones in which the degenerative replacement process had been the most active in the preceding years.

### Disease activity as estimated by elevated water T2 in the lower limb of Pompe patients

**Fig. 3:**
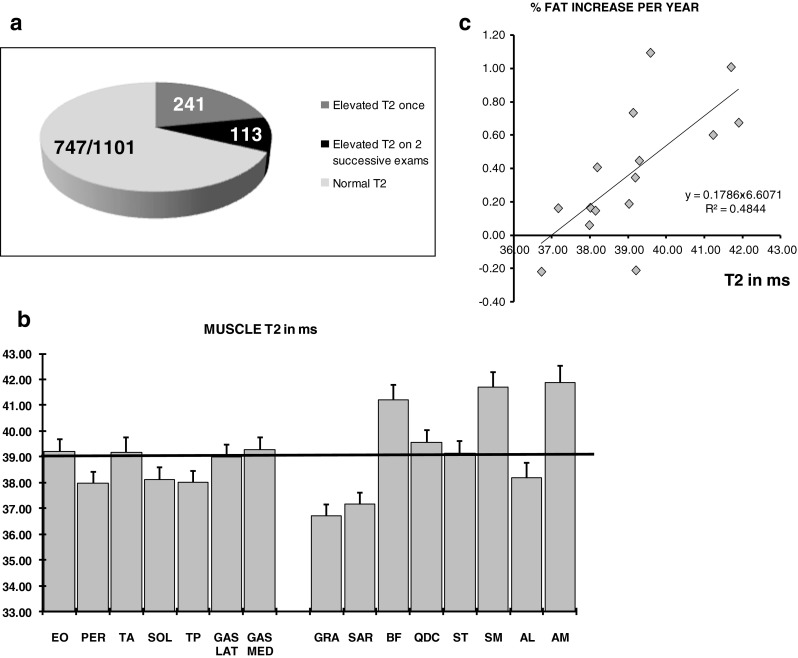
A high percentage of muscles, 32 %, were found with an abnormally high T2 in at least one of two successive imaging sessions as displayed by the pie-chart in A. Mean T2s for individual muscles are given in B, with the horizontal line at 39 ms representing the abnormality threshold. The adductor magnus, semi-membranous and biceps femoris had the highest disease activity. Mean muscle T2 was correlated with the mean annual increase in fat content, as shown in C

Of the 1101 muscles that were analyzed, 241 had abnormally high muscle water T2 (>39 ms) in at least one of two successive scans, and 113 had elevated T2 on both occasions. This amounted to 32 % of all investigated muscles displaying signs of disease activity in at least one of two successive follow-up examinations. Individual muscle analysis again revealed important differences across muscles (Fig. 3). Muscles that were systematically found with abnormal water T2 were all located in the thigh: the biceps femoris, the semi-membranous and the adductor magnus.

### Link between disease activity as revealed by high T2 and muscle degenerative progression as measured by the fatty infiltration rate

When muscle water T2 was abnormal, the progression rate of fatty infiltration was 0.61 %/year higher than when T2 was found within the normal range (*p* = 0.02). Muscles having a higher T2 were muscles experiencing on average the faster progression of the fatty degenerative changes (Fig. 3c).

### Effect of enzyme replacement therapy on the progression of the lower limb degenerative changes

**Fig. 4:**
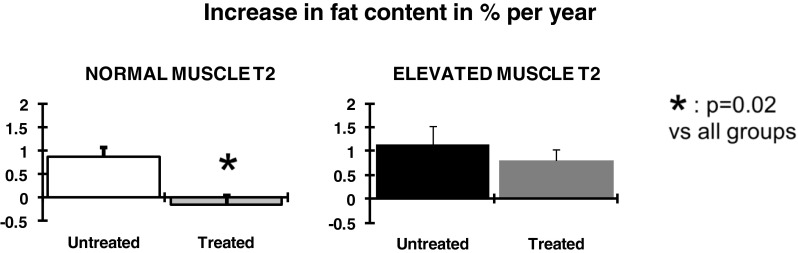
Effect on enzyme replacement therapy on the progression of the fatty degenerative changes. Treatment was effective both in muscle with normal T2 (*left panel*) and with elevated T2 (*right panel*). In muscles with normal T2s, the progression of the degenerative changes was completely blocked

In muscles of patients who were benefiting from enzyme replacement therapy with alglucosidase alfa, the yearly progression rate of fatty infiltration rate was lower by 0.68 %/year as compared to the untreated patients (*p* = 0.01). In muscles with normal water T2, muscle degenerative changes were completely stopped (Fig. 4).

## Discussion

This work demonstrates that quantitative NMR (qNMR) imaging techniques are able to monitor disease progression in skeletal muscles of patients with low-activity dystrophic lesions, as here in late-onset Pompe disease.

### Visual scoring versus quantitative analysis of muscle imaging data

Routine diagnostic T1w MRI of muscle disorders makes uses of visual grades, typically from 0 or 1 for normal to 4 for full replacement by fat, to characterize the extent of the degenerative changes on a per muscle basis (Lamminen 1990). This is largely sufficient to characterize the patterns that will potentially orient toward a particular causative gene. However, common sense tells us that, with these so called “semi quantitative” scales, progression from one class to the next requires on average 12.5 or 16.5 % increase in muscle fat content. This is a most unlikely event over a 1- or 2-year follow-up, even in the most severe of muscle dystrophies. Here, in a much milder form of dystrophy, it was virtually foretold that the Lamminen-Mercuri scales would not be able to detect muscle degenerative changes, just as they previously failed to identify progression in an LGMD2I patient longitudinal study (Willis et al 2013).

The T2w STIR sequences so popular among clinicians and radiologists do not suffer from any similar sensitivity issue, but the problem lies in the qualitative nature itself of the information generated by such sequences. One looks visually for contrast between abnormal and normal muscles or areas. If all muscles in a segment are inflamed, the exam is likely to be considered falsely negative. Such situation was encountered in juvenile dermatomyositis patients (Carlier et al 2013). However, in a population like the Pompe patients, the major shortcoming of STIR sequences is the inability to objectively compare different patients or successive exams of the same patients in a longitudinal study.

Awareness of these limitations made us turn to carefully selected qNMR sequences for the longitudinal characterization of skeletal muscle involvement in this Pompe patient population. The low values of the progression of alterations which were detected between routine yearly follow-up justified this decision a posteriori but also demonstrated the power of qNMR approaches as compared to routine MRI when, beyond purely diagnostic questions, it comes to disease characterization.

### Monitoring of muscle degenerative changes with quantitative muscle imaging

Quantitative water-fat imaging using a Dixon sequence was able to detect skeletal muscle fatty degenerative changes progression, which was overall very small, less than 1 %/year in our population. This demonstrates the excellent sensitivity of the technique and confirms results recently published in a multi-center trial aiming at monitoring the structural changes in lower limb muscles of LGMD patients of type 2I (Willis et al 2013). The fat deposition rate, which was already slow in that population with an average of approximately 2 %/year, was even lower here in adult Pompe patient but was unambiguously captured. This confers quantitative Dixon sequences a unique value as a biomarker of the skeletal muscle structural changes in NMD patients.

Similarly to the LGMD2I study, important inter-muscle differences were noted, here with the fastest degradation recorded in the hamstring and in the thigh adductors. The significance and validity of these individual differences was confirmed by the strong correlation between the fatty infiltration progression rate and the severity of muscle involvement, the muscles with the fastest degenerative changes being the ones with the highest fat content. Since all muscles were initially fat-free, this had logically to be the case at least until sub-total fat replacement occurred.

Such benchmarking was important because it might increase the discriminative power of muscle imaging outcome measures by a selection of the most appropriate targets.

Dixon sequence parameters were selected to generate the highest proton-density weighing while maintaining an acceptable signal-to-noise ratio and therefore to provide fat percentage parametric maps that were as close as possible to the real hydrogen atom distribution between water and lipids.

Fibrosis is the other main chronic structural change that may develop in skeletal muscles of NMD patients. Attempts to evaluate interstitial fibrosis with standard NMR or Dixon imaging sequences have so far been inconclusive. Optimized B1 transmitter and receiver field compensation might in the future be able to reveal the drop in NMR signal intensity that ought to be associated with the presence of fibrotic tissue. This was not implemented in this study and therefore no information on abnormal connective tissue deposition was obtained. So far, the most promising sequence to quantify fibrosis are the ultra-short TE (UTE) sequences, that are not yet routinely used.

### Evaluation of “disease activity” with quantitative muscle imaging

Contrary to global T2 (Carlier 2014), muscle water T2 of fatty infiltrated muscle is a non-specific but sensitive index of what may be called “disease activity” (Carlier et al 2013). Despite the slowly progressive nature of late onset Pompe disease, the percentage of muscle displaying abnormally high T2 values was surprisingly almost one third in our population sample. The observation was all the more unexpected, that significant inflammatory reaction is most often absent at histological examination of Pompe biopsies. It can then be hypothesized that abnormal T2 could then rather reflect myocytic lesions, possibly in relation with lysosomal rupture. A link between high T2 and glycogen accumulation has sometimes been suggested. This seems unlikely. The T2 deconvolution experiments by Saab et al have, on the contrary suggested that water in close interaction with glycogen had shorter T2s than free water (Saab et al 2000). Also, McArdle patients who systematically have much higher glycogen content (Jehenson et al 1991) than Pompe patients show only a slight increase in muscle T2s (de Kerviler et al 1991). These evidence make a direct role of glycogen in the water T2 changes very improbable. It must also be recognized and stressed that the muscle T2 elevation noted in Pompe was of much lower amplitude than it can be in inflammatory myopathies, which is potentially compatible with the minimal degree of inflammation in Pompe and a more direct role of subtle myocyte lesions per se.

Muscles with abnormal T2s belong to the thigh, and mainly to the posterior segment, which are also the muscles of the lower limb in which the degenerative changes are classically most prevalent. Since a bias introduced by fat T2 can be ruled out with the processing method we use, this observation supports the contention that abnormal T2 might reveal disease aggressiveness. Additional evidence of this is discussed in the next paragraph.

### Link between disease activity and progression of degenerative changes

In patients suffering from fascio-scapulo humeral dystrophy (FSHD), it was shown that muscles with hyperintensities on T2w images would subsequently display significant fatty degenerative changes on later follow up scans (Friedman et al 2013). The future usefulness of T2 as a NMR biomarker in neuro-muscular therapeutic trials will depend on the solidity of the evidence in favor of such a predictive role.

Muscle of Pompe patients with abnormal water T2 experienced a faster progression of the degenerative changes. The absolute increment was small, but it could not have been otherwise with such an intrinsically slow progression of the disease. In relative terms however, an elevated water T2 implied almost a doubling of the fatty infiltration rate. Such an observation is pivotal in establishing the utility of muscle water T2 as a biomarker of disease activity in NMDs.

Also, anatomical segregation of muscles revealed a loose but significant correlation between mean muscle T2 and mean rate of progression of fatty infiltration, further supporting the predictive value of water T2. Fluctuations of T2 during the course of the disease might have impacted on the correlation, T2 being more an instant picture of the disease activity while fat percentage mapping provides more of an integrator of global disease burden over time. This explains why the two NMR variables are both of importance and complementary to assess consequences of the disease on muscles.

### Effect of treatment

In this open label retrospective study, the decision to initiate treatment had been taken by the physicians in charge according to the patient’s clinical presentation. As a result, the extent of the degenerative changes was significantly more severe in the patients receiving the enzyme replacement therapy (Lamminen-Mercuri 2.4 ± 0.2 in treated patients vs 1.5 ± 0.3 in untreated patients, *p* < 0.01). Still it was possible to compare the progression of the structural changes in muscles of treated versus untreated patients. We assumed that response to treatment would be independent of the initial degree of muscle involvement, which is not guaranteed. Nonetheless, if such bias existed here, if anything, it would likely have made the demonstration of a-glucosidase effect more problematic because of the more advanced lesions in the treated group.

Data gathered over the last decade now unambiguously show that enzyme replacement therapy improve life expectancy of late onset Pompe patients (Güngör et al 2013). Evidence of functional improvement was far less spectacular with a modest increase in the 6MWT distance and no change in pulmonary FVC (Lachmann and Schoser 2013). Effect of treatment on muscle structure has even been more difficult to prove, despite apparent clearance of glycogen (Thurberg et al 2006). An increase in muscle trophicity of 3 to 8 % over 6-month of treatment was nicely identified by a quantitative analysis of MRI data. It was surprisingly associated with a comparable accumulation of subcutaneous fat (Ravaglia et al 2010).

In the present report, enzyme replacement therapy resulted in a lesser percentage of intramuscular fat. The effect is modest but highly significant and the overall yearly accumulation of intramuscular fat was small anyhow. A bit surprisingly, the decrease in percentage fat signal was identical in muscles with normal and high T2s and treatment was not associated with a decrease in muscle T2. The mechanisms responsible for the decrease in fat fraction accumulation rate under enzyme replacement therapy are to be further elucidated. Whether it might or not, partly or totally, reflect indirectly an increase in muscle mass is a possibility that has to be explored.

### NMR vs other outcome measures

The comparison of the qNMR data with the other and mainly the functional outcome measures was beyond the scope of this report and has yet to be performed. Studies have unambiguously demonstrated the superiority of qNMR over routine functional assessment to document disease progression in other diseases. It was the case in a longitudinal multicentric natural history study of LGMD2I adult patients (Willis et al 2013). It was similarly shown in the large pediatric survey of young Duchenne muscular dystrophy patients in the US (Willcocks et al 2014).

## Conclusion

This retrospective monocentric study demonstrated the value of qNMR imaging in the context of a longitudinal monitoring of muscle lesions in adult Pompe patients and provided unambiguous answers to our questions:

- quantitative water-fat imaging of skeletal muscle can assess the slow progression of the fatty degenerative changes in muscles of adult Pompe patients;
- up to one-third muscles of the lower limbs show moderate signs of disease activity revealed by high water T2 in these patients;
- disease activity, as measured by T2 maps, is a predictor of progression of the fatty degenerative changes in this population;
- enzyme replacement therapy can limit the progression of muscle damages in adult Pompe patients.

## Electronic supplementary material

Below is the link to the electronic supplementary material.

ESM 1 (DOCX 13 kb)
