# Supplementary material for: Skeletal muscle quantitative nuclear magnetic resonance imaging follow-up of adult Pompe patients
Source: J Inherit Metab Dis. 2015 Mar 7;38(3):565–72. doi: 10.1007/s10545-015-9825-9 (PMC4432102; doi:10.1007/s10545-015-9825-9)
Supplement: Supplementary file 1 — (DOCX 13 kb) [file 10545_2015_9825_MOESM1_ESM.docx]

Supplementary Table : Population demographics

Untreated patients Treated patients

Age (in yrs, + SD) 50, +12 47, +11

Male/female (n/n) 3M/6F 3M/11F

Body mass index (kg/m², + SD) 24.47 +3.55 24.43 +3.31

Ambulation (n=) 9 14

Nocturnal ventilator assistance (n=) 0 0
